# *CADM1 *is a strong neuroblastoma candidate gene that maps within a 3.72 Mb critical region of loss on 11q23

**DOI:** 10.1186/1471-2407-8-173

**Published:** 2008-06-17

**Authors:** Evi Michels, Jasmien Hoebeeck, Katleen De Preter, Alexander Schramm, Bénédicte Brichard, Anne De Paepe, Angelika Eggert, Geneviève Laureys, Jo Vandesompele, Frank Speleman

**Affiliations:** 1 Center for Medical Genetics, Ghent University Hospital, Ghent, Belgium; 2 Department of Pediatric Oncology and Hematology, University Children's Hospital of Essen, Essen, Germany; 3 Department of Pediatric Hematology and Oncology, Cliniques St Luc, Université Catholique de Louvain, Brussels, Belgium; 4 Department of Pediatric Hematology and Oncology, Ghent University Hospital, Ghent, Belgium

## Abstract

**Background:**

Recurrent loss of part of the long arm of chromosome 11 is a well established hallmark of a subtype of aggressive neuroblastomas. Despite intensive mapping efforts to localize the culprit 11q tumour suppressor gene, this search has been unsuccessful thus far as no sufficiently small critical region could be delineated for selection of candidate genes.

**Methods:**

To refine the critical region of 11q loss, the chromosome 11 status of 100 primary neuroblastoma tumours and 29 cell lines was analyzed using a BAC array containing a chromosome 11 tiling path. For the genes mapping within our refined region of loss, meta-analysis on published neuroblastoma mRNA gene expression datasets was performed for candidate gene selection. The DNA methylation status of the resulting candidate gene was determined using re-expression experiments by treatment of neuroblastoma cells with the demethylating agent 5-aza-2'-deoxycytidine and bisulphite sequencing.

**Results:**

Two small critical regions of loss within 11q23 at chromosomal band 11q23.1-q23.2 (1.79 Mb) and 11q23.2-q23.3 (3.72 Mb) were identified. In a first step towards further selection of candidate neuroblastoma tumour suppressor genes, we performed a meta-analysis on published expression profiles of 692 neuroblastoma tumours. Integration of the resulting candidate gene list with expression data of neuroblastoma progenitor cells pinpointed *CADM1 *as a compelling candidate gene. Meta-analysis indicated that *CADM1 *expression has prognostic significance and differential expression for the gene was noted in unfavourable neuroblastoma versus normal neuroblasts. Methylation analysis provided no evidence for a two-hit mechanism in 11q deleted cell lines.

**Conclusion:**

Our study puts *CADM1 *forward as a strong candidate neuroblastoma suppressor gene. Further functional studies are warranted to elucidate the role of *CADM1 *in neuroblastoma development and to investigate the possibility of *CADM1 *haploinsufficiency in neuroblastoma.

## Background

Neuroblastoma (NB) is a rare but often highly aggressive tumour in children. Despite intensive gene copy number and mRNA expression studies thus far only two genes, namely *MYCN *[1]and *PHOX2B *[2-4] have been found to be directly implicated in NB development. In order to provide clues for more effective therapies, insights into the molecular pathogenesis of this tumour are urgently needed. Analyses of recurrent patterns of somatically acquired DNA copy number alterations resulted in the delineation of three major genetic subgroups with predictive tumour behaviour (subtype 1, 2A and 2B), of which subtype 2A NB represents an aggressive subgroup of metastatic NB [5,6] characterised by loss of 11q, gain of 17q and a normal *MYCN *copy number status [6].

Deletion of the long arm of chromosome 11 is found in 15–22% of sporadic NB [5-10] and has also been described in constitutional cases of NB [11,12], suggesting the presence of one or more tumour suppressor gene(s) on chromosome 11. Functional evidence for a NB tumour suppressor gene at 11q came from microcell mediated chromosome transfer (MMCT) experiments, in which transfer of an intact chromosome 11 in a NB cell line with 11q loss resulted in a more differentiated phenotype [13]. Array comparative genomic hybridisation (array CGH) analysis of these MMCT hybrids revealed 11q25 as a plausible location for a NB differentiation gene [14]. Extensive microsatellite heterozygosity mapping studies however point at various critical regions of loss, located at 11q23.3 [7] and within the chromosomal region 11q14-11q23 [15]. Despite these mapping efforts, no genes with proven tumour suppressor activity in NB have been identified thus far.

In this study, we used an integrated approach combining high resolution copy number profiling and gene expression meta-analysis. This approach identified *CADM1 *as a strong candidate 11q tumour suppressor gene with prognostic power, which possibly exerts its effect through haplo-insufficiency.

## Methods

### Array CGH copy number profiling of NB patients and cell lines

A description of the primary NB tumour samples and NB cell lines as well as the array CGH procedure is given in Michels et al. [16]. Twenty-five additional tumour cases were profiled for this study, including 4 stage 1, 3 stage 2, 6 stage 3, 9 stage 4 and 3 stage 4S tumours according to the International Neuroblastoma Staging System [17]. The maximal size of the lost region is determined as the distance between the two normal clones flanking the lost clones. Detailed data for 75 tumours are published by Michels et al. [16]. Detailed data for the 25 additionally profiled tumour cases are available in Additional File 1. Data for all tumours are also accessible from the webtool arrayCGHbase [18,19]. Mapping data are based on Ensembl v44.

### Meta-analysis of published NB gene expression datasets

Expression data were collected from seven independent gene expression studies in NB [20-26]. For each of the individual expression studies Cox regression analysis (enter method) was performed for each gene present in the defined SROs using the SPSS 15 software, allowing to pinpoint genes within our SROs having prognostic power. Next, in four studies for which genomic data were available [21,22,25,26], tumours were also classified according to the three major NB subtypes: subtype 1 (characterised by mainly numerical aberrations and not 11q loss or *MYCN *amplification), 2A (characterised by 11q loss, without *MYCN *amplification) or 2B (characterised by *MYCN *amplification). Following this subclassification an independent Kruskal-Wallis analysis was performed for each of the four studies using the R software package with multiple testing correction. Genes were considered to be significant when a p-value < 0.5 was calculated (Additional file 2).

Following these analyses, an intersection was made between genes that were prognostically significant (Cox regression analysis) in more than three independent studies and genes which had subgroup discriminating power in more than two independent studies (Kruskal-Wallis analysis) to identify the strongest candidate genes (see results and discussion).

### mRNA expression analysis for *CADM1 *in cell lines untreated or treated with DAC

NB cells were plated at day 0 and treated 24 h later with 3 μM DAC during 3 days. The NB cell line panel consisted of CHP-134, CHP-901, CHP-902R, CLB-GA, GI-C-IN, IMR-32, LA-N-1, LA-N-2, N206, NBL-S, NGP, NLF, NMB, SH-SY5Y, SH-SY5Y TrkA, SJNB-1, SJNB-8, SJNB-12, SK-N-AS, SMS-KAN, SMS-KCNR and STA-NB-1.2. Cells were harvested and RNA was extracted for real-time quantitative PCR using the RNeasy Mini kit (Qiagen). Primers for *CADM1 *and four stably expressed reference genes (*HPRT1*, *GAPDH*, *YWHAZ*, *HMBS*) are available in the public RTPrimerDB database [27-29] (gene (RTPrimerDB-ID): *HPRT1 *(7), *GAPDH *(3), *YWHAZ *(7), *HMBS *(4) and *CADM1 *(3770)). DNAse treatment and real-time PCR analysis were performed as previously reported [30].

### Bisulphite sequencing

A cell line panel was composed including CHP-134, CHP-901, CLB-GA, GI-C-IN-1, LA-N-1, LA-N-5, NB-13, NB-19, N206, NBL-S, NGP, NLF, SHEP, SJNB-1, SJNB-6, SJNB-8, SJNB-10, SJNB-1.2, SK-N-AS, SKN-BE (1n), SK-N-BE (2c), SK-N-FI, SMS-KAN, SMS-KCNR, STA-NB-3, STA-NB-8, STA-NB-9, STA-NB-10, STA-NB-1.2, TR-14 and UHG-NP. Approximately 1 μg of DNA was modified with sodium bisulphite using the EZ DNA Methylation Kit (Zymo Research). The resulting modified DNA was used as a template in a PCR reaction. Briefly, PCR reaction was carried out in a 50 μl reaction containing 30 ng of bisulphite modified DNA, 1× Platinum Taq PCR reaction buffer (Invitrogen), 6 mM MgCl_2_, 200 μm of each dNTP, 1.25 U Platinum Taq polymerase (Invitrogen) and 300 nm of each primer. PCR cycling conditions were as follows: 4 min of initial denaturation at 93°C, followed by 40 cycles of 30 s denaturation at 93°C, 50 s of annealing at 57°C and 30 s of extension at 72°C, followed by a final extension step of 4 min at 72°C. Primers were developed using Bisearch [31,32] and tested for specificity using our in house developed methBLAST software [33] and are available in the public methPrimerDB database [33](ID 243). The primers were designed to assess the methylation status of 20 CpG sites situated upstream of the transcription start 4 μl of PCR product was cloned into TOPO-TA vector and transformed into *E. coli *TOP10F' cells (Invitrogen) and 10 positive clones were randomly selected for direct sequencing using universal M13 primers. As a control, normal human genomic DNA and SssI methylase (New England Biolabs) treated DNA were processed respectively as a negative and a positive control for methylation after bisulphite modification and evaluated for overall bisulphite conversion using sequencing. A 100% conversion was reached (data not shown). Furthermore, we verified for preferential amplification of unmethylated or methylated alleles through sequencing of artificial mixture samples from normal DNA and different concentrations of methylated DNA. We could confirm no amplification bias (data not shown).

## Results and Discussion

In an attempt to more accurately map 11q losses and to identify potential homozygous losses that were previously missed using low resolution analyses, we performed high resolution BAC array CGH copy number profiling on 100 primary NB tumours (16 stage 1, 12 stage 2, 21 stage 3, 40 stage 4 and 11 stage 4S tumours) and 29 cell lines using a 1 Mb BAC array supplemented with a chromosome 11q tiling path (position 63–72 Mb (11q13) and 84–134 Mb (11q14.1-qter)). Three patterns of chromosome 11 loss could be observed. Twenty-four (24%) tumours showed loss of the entire chromosome 11. These tumours consisted of predominantly low stage tumours with an overall pattern of numerical imbalances. In addition, also 3 cell lines (10%) showed whole chromosome 11 loss. Seventeen tumours (17%) and 16 cell lines (55%) showed partial 11q loss. Most deletions were large encompassing the distal end of 11q, in keeping with data from literature [6-10,15,34,35]. In cell lines CLB-GA, SMS-KAN, and NB-5, small interstitial losses were detected (respectively minimal 42.90, 30.84, and 4.76 Mb in size). To our knowledge this is the first report of interstitial 11q loss in SMS-KAN and NB-5 cell lines. No homozygous 11q losses were observed.

**Figure 1:**
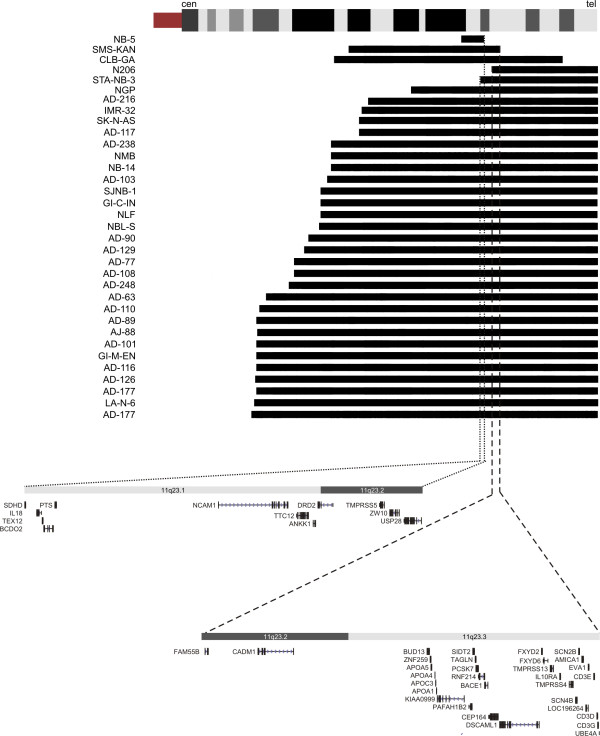
**Array CGH copy number profiling of chromosome 11 in neuroblastoma**. **Top**. Alignment of array CGH profiles for 17 primary tumours and 16 cell lines showing partial 11q loss. Copy number losses are indicated by black bars along the chromosome 11q ideogram (cen= centromere, tel= telomere). The common regions of loss are marked by dotted bars (SRO 1: dashed line, 3.54–3.72 Mb and SRO2: dotted line, 1.65–1.79 Mb).**Bottom**. Detailed view of genes within the two putative SROs.

Two common minimal regions of loss (shortest region of overlap, SRO) at 11q23 could be delineated (Figure 1). The telomeric SRO (SRO1, 3.54–3.72 Mb, 11q23.2-q23.3) is delineated by the breakpoints in N206 (centromeric border) and SMS-KAN (telomeric border) and contains 31 genes. More centromeric, a second SRO (SRO2, 1.65–1.79 Mb, 11q23.1-q23.2) is delineated by the breakpoints in STA-NB3 (centromeric border) and NB-5 (telomeric border) and contains 12 genes. Recent evidence indicates that also miRNAs can function as tumour suppressor genes. According to the miRBase, [36,37] (August 2007, version 10.0) no miRNAs were however located within the defined SROs. The finding that the most distal SRO partially overlaps with the SRO defined by Guo et al. [7] and both SROs are positioned within the critical region defined by Maris et al. [15] validates our mapping efforts. Nevertheless, it is important to note that SRO mapping should always be interpreted with care as individual observations can dramatically reduce the size or location of the SRO. This is especially the case when including cell line data, in which alterations could be induced during culturing. In this respect, it should therefore be noted that NB cell lines have been proven to be very stable over time and that, although rare, interstitial 11q loss has also been described in primary NB tumours [6,7,15,38], further underscoring the relevance of our defined SRO regions.

To prioritize potential NB 11q candidate genes within the two critical regions, we performed a meta-analysis on publicly available gene expression data of 692 tumours from seven independent studies [20-26]. An overview of the obtained results is given in Additional file 2. In a first analysis we determined which genes in our SROs have prognostic power using Cox regression analysis. In total, 24 genes were prognostically significant in one or more studies (see Additional file 2), of which four *(CADM1, NCAM1, CD3E, ZNF259) *were significant in three or more studies. In the four studies for which DNA copy number data were available, we determined in a second analysis which genes could discriminate between the various NB subgroups (subtype 1, 2A, 2B) (Kruskal-Wallis test). This multi-group analysis was specifically applied instead of a binary comparison between 11q normal versus 11q deleted samples in order to avoid combining tumours with a distinct biology and prognosis. Thirteen genes had discriminative power, of which five were significant in at least two studies *(CADM1, NCAM1, BACE1, ZNF259, KIAA0999)*. Genes that were prognostic significant in more than three independent studies and had subgroup discriminating power in more than two independent studies were considered as strong candidate genes (*CADM1, NCAM1 *and *ZNF259)*.

*ZNF259 *is a zinc finger protein involved in normal cell cycle progression [39]. *ZNF259 *has been suggested as a modifier gene in spinal muscular atrophy [40], but no direct link with carcinogenesis has been established thus far. *NCAM1 *is a neural cell adhesion molecule with an important role in tumour migration and metastasis. In a rat model it was demonstrated that *NCAM *transfected glioma cells show a decreased invasion compared to low *NCAM *expressing control cells [41]. Similarly, loss of *NCAM *in a transgenic mouse model of pancreatic β cell carcinogenesis caused the formation of lymph node metastasis, whereas *NCAM *overexpression prevented tumour metastasis [42]. In several tumour types, including colon carcinoma [43], gastrointestinal neoplasia [44] and astrocytic tumours [45], downregulation of *NCAM *expression and an associated poor survival has been described. In chemoresistant NB cells an enhanced invasive capacity was observed due to downregulation of *NCAM *adhesion receptors [46]. *CADM1 *encodes a cellular adhesion molecule with a role in synaptic formation of neural cells [47]. This gene, also known as *IGSF4 *(immunoglobulin superfamily, member 4) or *TSLC1 *(tumour suppressor in lung cancer-1), is a *bona fide *tumour suppressor gene in non-small lung cancer and was identified by functional complementation of a frequently deleted region in nude mice [48]. Reduced or lost expression has been described in a variety of human cancers and *in vivo *suppression of tumorigenesis after restoration of expression has been demonstrated in esophageal cancer, cervical cancer and prostate cancer [49-52].

To select the most promising candidate gene within the three remaining candidate genes, we integrated the gene expression profile of normal NB precursor cells (see Additional file 2). The transcriptome information of these normal fetal neuroblasts represents a unique resource for identifying genes contributing to NB development and the malignant phenotype [21]. Remarkably *CADM1 *was the only gene in our candidate list that was differentially expressed in NB versus neuroblasts using the Rank Product algorithm. A significantly lower expression of *CADM1 *was observed when comparing unfavourable NB to neuroblasts [21]. This is in agreement with the Kruskall-Wallis data showing *CADM1 *downregulation in unfavourable NB compared to favourable subtype 1 NB. Consequently, we consider *CADM1 *to be a compelling candidate NB tumour suppressor gene for further analysis.

**Figure 2:**
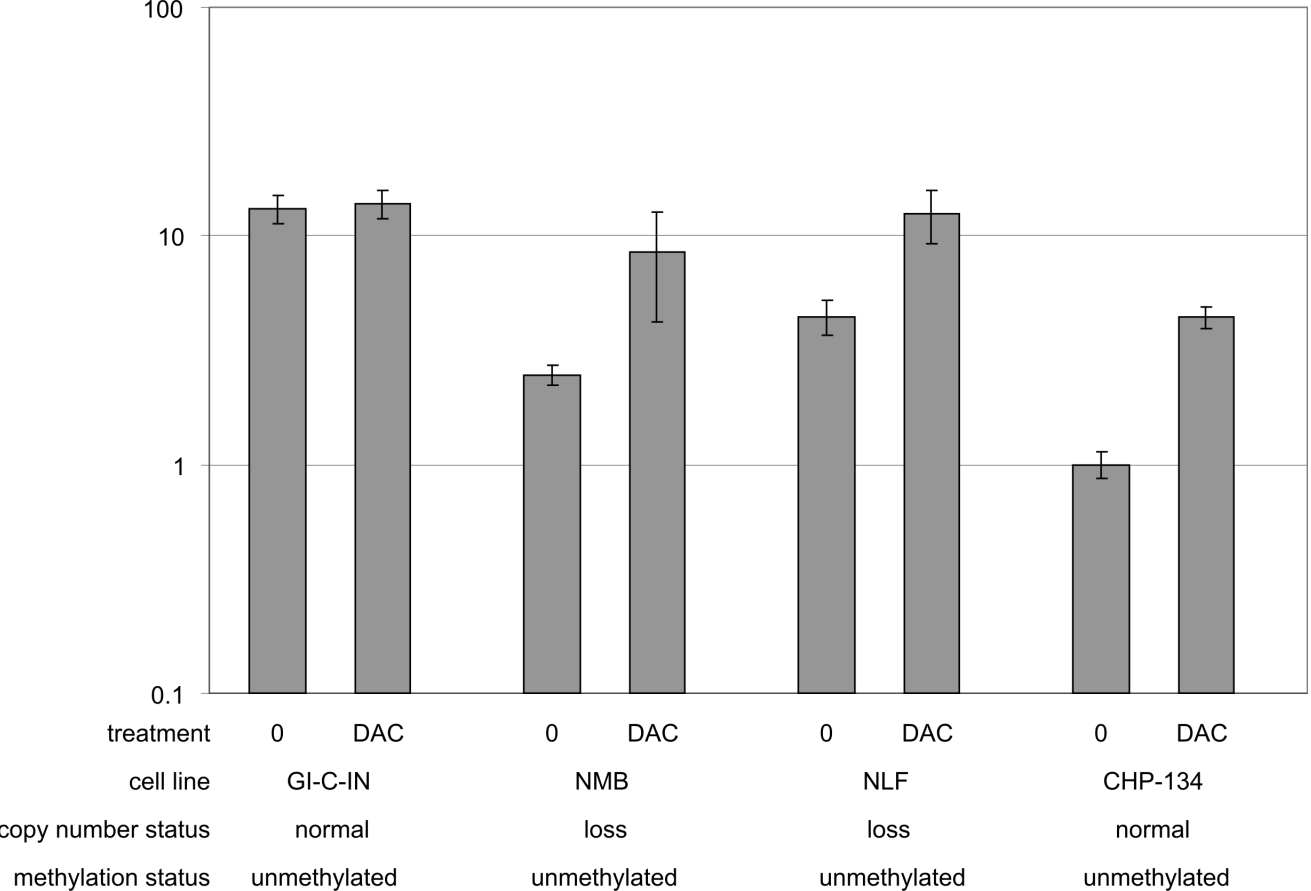
**Representative examples of *CADM1 *mRNA expression in cell lines untreated (0) or treated with 5-aza-2'-deoxycytidine (DAC)**. Cell lines NMB, NLF and CHP-134 show a more than 2-fold upregulation of *CADM1 *expression after treatment with DAC. *CADM1 *copy number status is indicated: NB cell lines GI-C-IN and CHP-134 show no loss of the *CADM1 *locus as determined by array CGH, while in NMB and NLF loss of *CADM1 *was observed. All cell lines were unmethylated following bisulphite sequencing. Lowest expression is rescaled to 1.

In several cancer types, two-hit inactivation of *CADM1 *occurs by LOH and hypermethylation of the promoter region, whereas *CADM1 *mutations are rare. Indeed, it was shown that only two *CADM1 *sequence variants could be picked up in a panel of 31 NB cell lines [53], while in a study of 25 NB with proven *CADM1 *allelic loss no mutations were found in the remaining allele [54]. Epigenetic modification has been described for several genes in NB [55,56]. To study if the remaining allele is subject to methylation and resulting epigenetic silencing, we measured *CADM1 *mRNA expression in a panel of 22 NB cell lines before and after exposure to the demethylating agent 5-aza-2'-deoxycytidine (DAC). Four out of 13 cell lines with loss at the *CADM1 *locus showed a more than two fold upregulation of *CADM1 *mRNA expression after DAC treatment. This was also the case for two of the nine cell lines with normal *CADM1 *copy number. Representative examples of mRNA expression before and after treatment with DAC are given in Figure 2. As the maximal observed expression upregulation was 4.4 fold in cell line CHP-134, these data were inconclusive to predict the involvement of methylation associated *CADM1 *silencing in NB. Consequently, we performed bisulphite sequencing of twenty promoter region CpG dinucleotides in a panel of 31 NB cell lines (including the cell lines that were studied in the DAC experiment). Note, that the investigated CpG sites also cover the 93 bp consensus CpG region described by Kuramochi et al. [48]. Methylation of all studied CpG sites was identified in 12.5, 20 and 11% of analysed clones in the cell lines NGP, SH-EP and SK-N-BE(2c). It should be noted that NGP was also present in the panel of cell lines treated with DAC, where a more than two fold upregulation was observed after DAC treatment. However, in all other cell lines only sporadic CpG methylation was detected at low frequency (< 30%). Based on this evidence and taken in account that cell lines typically show a higher extent of CpG island methylation compared to primary NB, epigenetic *CADM1 *inactivation probably does not play a major role in NB. The latter could explain the observed limited fold changes in the DAC re-expression data.

While *CADM1 *forms an excellent positional and functional candidate tumour suppressor gene for NB, we can not exclude a role for the 2 other identified candidates suppressor genes *ZNF259 *and *NCAM1*. Based on our own methylation and previously published mutation data, *CADM1 *is most likely not inactivated through a two-hit Knudson mechanism. However, novel concepts in tumour suppressor genetics such as haploinsufficiency currently challenge the two-hit paradigm and are gaining importance in cancer genetics. Hence, an intriguing possibility is sensitivity of *CADM1 *to gene dosage. In this respect, the observed reduced expression of *CADM1 *in unfavourable NB as compared to normal neuroblasts is interesting. *CADM1 *belongs to the family of immunoglobulin cell adhesion molecules and encodes a transmembrane glycoprotein involved in cell-cell interactions. As disruption or altering of cell-cell interactions is an important feature of tumour invasion and metastasis, it could therefore be hypothesised that one working *CADM1 *allele is insufficient to accomplish the normal gene function. This would make NB cells more aggressive and amenable for migration, invasion and metastasis formation. Furthermore, although the biological cascade of the CADM1 protein is not yet elucidated, it is interesting to note that *EPB41L3 *– an established binding partner of *CADM1 *[57] – has been described as higher expressed in mass screening NB tumours versus aggressive NB [58] and favourable NB versus unfavourable NB [21]. In 4/5 data sets, for which expression data on *CADM1 *and *EPB41L3 *were available, also a significant positive correlation between *CADM1 *and *EPB41L3 *mRNA expression was found (p < 0.05). Further research should therefore be performed to elucidate the involvement of *CADM1 *in NB.

## Conclusion

Tumorigenesis is a multistep process characterised by a multitude of genetic and epigenetic alterations. In view of the limited success of classical genomic approaches towards the identification of NB tumour suppressor genes, we integrated high resolution array CGH with transcriptome analysis and identified *CADM1 *as a candidate 11q tumour suppressor gene with prognostic power. *CADM1 *does not seem to be inactivated through a two-hit mechanism in NB. However, due to the frequent involvement of *CADM1 *in human tumorigenesis and its presumed role in invasion, a hallmark of malignancy, further studies should be performed to confirm *CADM1 *involvement in NB and elucidate its mode of action.

## Competing interests

The authors declare that they have no competing interests.

## Authors' contributions

EM carried out the genomic studies and drafted the manuscript. JH performed the methylation study. KDP carried out the meta-analysis of neuroblastoma mRNA expression data. GL, AS, AE and BB provided tumour material. JV and FS designed and coordinated the study, and edited the manuscript. All authors have reviewed the manuscript and FS, JV and ADP were the final editors of the manuscript.

## Pre-publication history

The pre-publication history for this paper can be accessed here:



## Supplementary Material

Additional File 1 Clinical characteristics of 25 additionally profiled primary NB tumours Click here for file

Additional file 2 **Meta-analysis on neuroblastoma gene expression data sets**. Meta-analysis on publicly available neuroblastoma gene expression data sets for genes residing in the two delineated critical regions of loss. Genes that were statistically significant in one or more studies are represented and ordered from centromere to telomere. Black boxes represent a significant p-value (<0.05) in the respective study. Grey boxes represent absence of a gene specific probe on the expression array (nb: normal neuroblasts, NB: neuroblastoma, F: favorable and UF: unfavorable prognosis according to De Preter et al., 2006 [21]). Click here for file
